# Investigating Male Tobacco Use and Expenditure Patterns across Socio-Economic Groups in Nigeria

**DOI:** 10.1371/journal.pone.0122021

**Published:** 2015-04-09

**Authors:** Nkoli P. Uguru, Chinyere Mbachu, Ogochukwu P. Ibe, Chibuzo C. Uguru, Oluwakemi Odukoya, Chinenye Okwuosa, Obinna Onwujekwe

**Affiliations:** 1 Department of Preventive Dentistry, Faculty of Dentistry, College of Medicine, University of Nigeria, Enugu Campus, Enugu, Nigeria; 2 Department of Community Medicine, College of Medicine, University of Nigeria, Enugu Campus, Enugu, Nigeria; 3 Health Policy Research Group, Department of Pharmacology and Therapeutics, College of Medicine, University of Nigeria, Enugu Campus, Enugu, Nigeria; 4 Department of Oral and Maxillofacial Surgery, Faculty of Dentistry, College of Medicine, University of Nigeria, Enugu Campus, Enugu, Nigeria; 5 Department of Community Medicine, College of Medicine, University of Lagos, Lagos, Nigeria; 6 Department of Health Administration and Management, Faculty of Health Sciences and Technology, College of Medicine, University of Nigeria, Enugu Campus, Enugu, Nigeria; Medical University Vienna, AUSTRIA

## Abstract

The magnitude of variation in economic costs of tobacco consumption among socio-economic status (SES) groups in Nigeria is unclear. Understanding the factors that influence tobacco use and expenditure among different socio-economic groups would inform decisions on interventions for tobacco control in Nigeria. Secondary data was obtained from the 2008 National demographic and health survey. Information on tobacco use and expenditure in households and individual males were extracted from the database. A total of 34,070 households and 15,846 individual males were sampled. Analysis was done using descriptive statistics and binary logistic regression analysis. Information on wealth index obtained were categorized into socio-economic quintile groups (Q1 to Q5), representing poorest to richest socio-economic groups. To estimate expenditure on cigarettes, the average cost of a stick of cigarette was obtained and multiplied with the number of sticks smoked per day. The proportion of households that use tobacco in Nigeria is 5.25% with a greater percentage (89.6%) residing in the rural areas. Prevalence of cigarette smoking in individual males is 8.59%, and the poorer SES group smoked more cigarettes (20.9%) and spent more (0.60–1.19USD) than the richest SES group. Low education level, traditional beliefs, literacy levels, SES and employment status all influence cigarette smoking in adult males. Although poor people smoked more and spent more of their income on cigarettes, other factors like educational level and traditional beliefs were found to influence practice of cigarette smoking in men. This implies that tobacco control legislation through increased taxes alone may not effectively reduce the use of tobacco and its products in Nigeria. A consolidated approach that includes behavioral change procedures, enforcing bans on tobacco advertisement and the use of strong graphic anti-tobacco messages targeted at both the poor and rich as well as the educated and uneducated need to be effected to reduce tobacco use.

## Introduction

Nigeria consumes about 20 billion sticks of cigarette which are valued at 200 billion Naira annually [1]. This can be related to the fact that Nigeria is no longer just an importer but also a net producer and exporter of tobacco products [1]. Tobacco companies argue that tobacco sales have a positive impact on the economy ignoring the individual costs borne by the citizens as a consequence of tobacco use [1]. Elsewhere, smoking related expenses has been found to have pushed a significant proportion of low-income families into poverty [2] and in Nigeria where about 70.8% live on less than a dollar per day [3], money spent on tobacco is money not spent on basic necessities such as food, shelter, education and health [3]. Thus the economic costs of tobacco consumption in Nigeria might vary among people of low, middle and high-income earnings however [3], the magnitude of this variation is not clear.

There have been several attempts to legislate tobacco dating back to 1990 with the establishment of the Tobacco Smoking (Control) Decree 20, 1990. With the transition from military to democratic governance in 2001, the decree was converted to an act titled “Tobacco (Control) Act 1990 CAP, T 16”. Under the provisions of the act, smoking in specific places such as schools and stadia were banned. It also required warning messages on all tobacco and sponsorship advertisement. The warning, “The Federal Ministry of Health warns that smokers are liable to die young,” resulted from the enforcement of the act but the ban on smoking in the specified public places was not enforced and so was ineffective. Since 1990, the country has been commemorating “World No Tobacco Day” (WNTD). This was previously organized by civil society groups, who created awareness and disseminated information to the public on the dangers of tobacco use, the FMOH began organizing WNTD events in the late 2000s. In 1999, the Federal Government re-vamped its position on tobacco control by inaugurating a National Smoking Cessation Committee that developed a short-term plan action. This culminated in a total ban on tobacco advertising by the Advertising Practitioners Promotion Council of Nigeria (APCON) in 2002 [4].

Increased awareness about the dangers of smoking and stricter tobacco control measures in the past two decades have led to a decline in the number of smokers in some developed countries [1]. However the inability of Nigeria to apply these same strict control measures point to a less promising decline in the number of smokers in the country. In 2001 the Nigerian government signed a memorandum of understanding with British America Tobacco to build a state of the art tobacco factory which has had a profound effect on tobacco in Nigeria from the growing of the leaf through to the manufacturing and distribution of tobacco products. Then in 2004, Nigeria signed the WHO Framework Convention for Tobacco Control (FCTC) and ratified it on the 20th of October, 2005. In June 2006, the Honorable Minister of Health inaugurated a multi-sectorial/inter-ministerial committee on tobacco control in Nigeria [4]. Since Nigeria is one of the African countries that signed the WHO’s Framework Convention for Tobacco Control, she is obliged to adopt and implement effective legislation aimed at reducing tobacco use and tobacco smoke exposure [1]. However, the National Tobacco control bill which was passed in March, 2011 and has since then passed its second reading at the Nigerian House of Representatives is yet to be signed into law [1].

In order to appreciate the extent of expenditures on tobacco products and the possible implications for the poorest, it is important to quantify the amount of tobacco being used as well as the tobacco related expenditure. Thus evidence on household tobacco consumption and expenditure pattern in Nigeria could provide useful information for policy-makers and possibly speed up the process of signing the bill into law. Understanding the factors that influence tobacco use among different SES groups could also inform decisions on interventions for tobacco control in Nigeria. This study therefore provides new information on tobacco use and expenditure, and possible determinants of use among different SES groups.

## Methodology

### Ethical Considerations

The data for the study was obtained from a population based survey from the 2008 National demographic health survey (NDHS). There were no identifiers linking the respondents to the data.

Permission to use the data was obtained from the National population commission in Nigeria.

### Study area

The data was obtained from the 2008 National demographic and health survey which was a cross sectional study conducted in Nigeria by the National population commission between the months of June and October 2008

#### Nigeria Country Profile

Nigeria is the most populous country in Africa with a 2011 population of 167 million (projected from 2006 Census, and a national growth rate estimated at 3.2 percent per annum [5, 6]. It lies on the West Coast of Africa between latitude 40 and 140 N and longitude 50 and 140 E. It occupies a land mass of approximately 923,768 square kilometers, sharing international borders with the Republics of Niger and Chad to the north, Cameroon to the east, Benin to the west, and the Atlantic Ocean to the south. The country operates a Federal system of government with the Executive, Judiciary, and a bicameral Legislative arm- the Senate and House of Representatives. The Federal Government of Nigeria (FGN) is headed by an elected President. Nigeria is made up of six geo-political zones, and administratively divided into 36 States. Each state is subdivided into local government areas (LGAs), and each LGA is divided into localities. In addition to these administrative units, during the 2006 Population Census, each locality was subdivided into convenient areas called census enumeration areas (EAs) [6]. Each state has an Executive, Judiciary, and a Legislative arm. The State Government is headed by an elected Governor and each LGA is governed by an elected Chairman and a Legislative Council. The Federal structure as outlined in the Nigerian Constitution provides for some level of administrative as well as financial autonomy of each State in the federation. Most of the country’s revenue is centrally generated and shared among the three tiers of government on an agreed revenue allocation formula. In addition, States and LGAs are autonomous and each generates independent internal revenues. Each tier of government prepares its own annual plan and budget [7].

### Sampling Method

The 2008 NDHS data was collected using a stratified two-stage cluster design. The primary sampling unit (PSU), referred to as a cluster for the 2008 NDHS, is defined on the basis of EAs from the 2006 EA census frame. The final survey clusters included 886 out of 888 census EAs (the other 2 could not be accessed due to flooding and inter-communal disturbances), and this served as the first stage of the sampling. A mapping exercise was carried out for each cluster by trained enumerators using Global Positioning System (GPS) receivers to obtain a complete list of households. The lists of households served as the sampling frame for the selection of households in the second stage. In the second stage of selection, an average of 41 households was selected in each cluster, by equal probability systematic sampling. A representative sample of 36,800 households was selected. In a sub-sample of half of the households all men age 15–59 years who were either permanent residents of the households in the 2008 NDHS sample or visitors present in the households on the night before the survey were eligible to be interviewed. Weighting was applied during sampling. The individual characteristics of the respondents were now elicited in this questionnaire and information on tobacco use was collected. The information on the wealth index was also elicited from the database.

### Data collection

Three questionnaires were used for the 2008 NDHS namely Household questionnaire, Women’s questionnaire and Men’s questionnaire. A careful study of these three questionnaires showed that the women questionnaire and data generated less than 1% of information on smoking and tobacco use so only data from the male and household questionnaires on tobacco use addressing the specific questions relevant to our topic were analyzed and used for this study. The Men’s Questionnaire was administered to all men age 15–59 in every second household in the 2008 NDHS sample. Information on tobacco use and expenditure in households was extracted from the database. Household consumption of tobacco was defined as the use of tobacco in any form by at least one member of the household. The primary respondent for the Household questionnaire, defined as the most knowledgeable member of the household, was asked if any member of the household was currently using tobacco in the following forms: smokes cigarettes, smokes pipe, chews tobacco, uses snuff, smokes other things and smokes nothing.

Consumption of tobacco by male household members was defined as the use of tobacco in any form by individual male members of the household. This information was collected using the Men’s questionnaire from male members of selected households who were ages 15–59 years. The questionnaire captured use of different forms of tobacco namely: smokes cigarettes, smokes pipe, chews tobacco, uses snuff, smokes other things; as well as the number of cigarettes smoked in the past 24 hours.

The wealth index of single figures was obtained from the categorization of the households into socioeconomic quintile groups using ownership of durable and nondurable goods; dwelling characteristics; type of drinking water source; toilet facilities and educational level. Each of these assets was assigned a factor score, generated through principal component analysis, and then standardized in relation to a standard normal distribution. Each household was then assigned a score for each asset, and the scores were summed for each household. Individuals were ranked according to the total score of the household in which they resided. The sample was then divided into quintiles from one (lowest) to five (highest). They are graded as follows Q1-poorest, Q2 = poorer, Q3 = Middle, Q4 = Richer, Q5 = Richest.

The WHO estimated cost of a pack of imported branded cigarettes in Nigeria as at 2008 ranged from 180 to 220 Naira. In order to estimate individual male house hold members’ expenditure on cigarette per day, the average cost of a pack of imported branded cigarette, which is 200 Naira, was used to calculate cost per stick [8]. This was then multiplied with the number of sticks smoked per day. Local brands and hand rolled cigarettes were not included.

### Data Analysis

SPSS version 17 and STATA version 10 statistical software were used to analyze the data which was self-weighted. Self–weighting was achieved by selecting clusters with probability-proportional-to-size (PPS) at all stages except the final stage, where a fixed number of households within a cluster were selected. Data from household questionnaire and male questionnaire were merged making sure that similar household identification numbers were used to match respondents in the two questionnaires. A total sample of 34,070 households was used in the analysis. Descriptive statistics was used to determine the proportion of households that use different forms of tobacco.

In order to identify the determinants of tobacco use, a binary logistic regression analysis was done. The set of independent variables tested were marital status, Literacy level, socio-economic status, employment status, regional differences, geographic differences (rural/urban), media exposure and age.

The wealth quintiles were cross tabulated with tobacco use to determine SES differences in tobacco use, the wealth quintiles were also cross tabulated with number of cigarettes smoked daily. This was then multiplied by average cost of a stick of cigarette to determine SES differences in daily cigarette expenditures.

### Strengths and Limitations

The NDHS data is nationally representative and this strengthens the applicability of the findings from this study in Nigeria as a whole, and in similar contexts. The study is however limited in scope because the authors had to rely on secondary data, which did not sufficiently explore other tobacco use-related variables of interest.

## Results

A total of 34,070 households and 15,486 individual male household members were used for data analysis, and the results are presented in Tables 1–5 below.

**Table 1:** Socio-demographic characteristics of respondents.

Socio-demographic characteristics of households (N = 34070)		
Variables	Attribute	N (%)
Type of place or residence	Urban	10724 (31.47)
	Rural	23346 (68.52)
Sex of head of household	Male	27852 (81.75)
	Female	6218 (18.25)
Wealth index	Poorest	7261 (21.31)
	Poorer	6735 (19.77)
	Middle	7214 (21.17)
	Richer	6800 (19.95)
	Richest	6060 (17.78)
Selection for men’s survey	Selected	16688 (48.98)
	Not selected	17382 (51.02)
Socio-demographic characteristics of males (N = 15486)		
Type of place of residence	Urban	5133 (33.15)
	Rural	10353 (66.85)
Religion	Christian	7907 (51.05)
	Islam	7254 (46.84)
	Traditional religion	215 (1.38)
	Other	110 (0.71)
Highest level of education	No education	3656 (23.60)
	Primary	3253 (21.00)
	Secondary	6490 (41.90)
	Higher	2087 (13.47)
Literacy*	Cannot read at all, blind, visually impaired	4279 (27.63)
	Able to read only parts of sentences	1555 (10.04)
	Able to read whole sentences	9558 (61.72)
	No card required with language**	94 (0.6)
Reads newspaper***	Not at all	8561 (55.28)
	Less than once a week	2740 (17.69)
	At least once a week	2832 (18.29)
	Almost everyday	1353 (8.74)
Listens to radio****	Not at all	1911 (12.34)
	Less than once a week	1409 (9.09)
	At least once a week	3280 (21.18)
	Almost everyday	8886 (57.38)
Watches television *****	Not at all	5939 (38.35)
	Less than once a week	2349 (15.16)
	At least once a week	2949 (19.04)
	Almost everyday	4249 (27.44)
Currently working	Yes	12859 (83.04)
	No	2627 (16.96)
Covered by health insurance	Yes	402 (2.60)
	No	15084 (97.40)

*Respondents were shown cards in major languages and asked to read whole sentences or parts of sentences.

**None of the reading cards were in a language the respondent could understand

***Those that were literate were asked if they could read newspaper

****includes those that do not listen at all to those who listen to radio almost everyday

***** includes those that do not watch television at all to those who watch to television almost everyday

**Table 2:** Tobacco use.

N = (34070)	Freq. (%)			p-value
Total number of households that use any form of tobacco	1787 (5.25)			0.00
Urban households	186 (10.40)			
Rural households	1601 (89.60)			
Males that use any form of tobacco (N = 15486)	1847 (11.90)			
*Different forms of tobacco use in males (N = 15486)	Total: n (%)	Urban: n (%)	Rural: n (%)	p-value
smokes cigarette	1331 (8.59)	424 (2.73)	907 (5.86)	0.29
smokes pipe	118 (0.76)	40 (0.26)	78 (0.50)	0.03
chews tobacco	100 (0.65)	24 (0.16)	76 (0.49)	0.05
uses snuff	512 (3.31)	111 (0.72)	401 (2.59)	0.00
Smokes other	112 (0.72)	39 (0.25)	73 (0.47)	0.71
SES differences among individual males that use tobacco				
SES group	Freq. (%)			p-value
Poorest (Q1)	418 (13.40)			0.00
Poorer (Q2)	366 (12.66)			
Middle (Q3)	421 (13.74)			
Richer (Q4)	373 (11.18)			
Richest (Q5)	269 (8.74)			

*Multiple responses

**Table 3:** SES differences in cigarette use and expenditure among male household members.

Variables	Poorest n[%]	Poorer n[%]	Middle n[%]	Richer n[%]	Richest n[%]	p-value
Cigarette use: N = 1331 (%)	278 (20.90)	253 (19.00)	286 (21.50)	296 (22.20)	218 (16.40)	0.02
**Number of sticks of cigarette/day (expenditure in USD)**						
0–9 (<0.60)	215 (77.30)	199 (78.70)	226 (79.00)	238 (80.40)	174 (79.80)	0.01
10–19 (0.60–1.19)	50 (18.00)	25 (9.90)	37 (13.00)	34 (11.50)	20 (9.20)	
20–29 (1.25–1.81)	11 (4.00)	14 (5.50)	13 (4.50)	13 (4.40)	13 (5.90)	
30–39 (1.87–2.43)	0	1 (0.40)	2 (0.70)	2 (0.70)	2 (0.90)	
40–49 (2.50–3.06)	0	0	2 (0.70)	0	1 (0.50)	
>50 (> 3.10)	2 (0.70)	14 (5.50)	6 (2.10)	9 (3.00)	8 (3.70)	

*1 stick of cigarette (cost of lowest brand tax inclusive) = ₦200 per pack of 20 @₦10 ($0.06) per stick^7^. [Currency exchange rate = 1dollar = 160 naira] (2012)

**Table 4:** Socio-demographic factors associated with cigarette smoking among individual males.

Variable	Socio-demographic factors	Proportion of male household members who smoke cigarette	p-value
Type of place of residence	Urban	424 (8.26)	0.29
	Rural	907 (8.76)	
Religion	Christianity	794 (22.46)	0.00
	Islam	469 (6.46)	
	Traditional religion	57 (26.51)	
	Other	11 (12.50)	
Literacy	Cannot read at all, blind, visually impaired	416 (9.92)	0.00
	Able to read only parts of sentence	155 (10.00)	
	Able to read whole sentence	744 (7.78)	
	No card with required language	9 (9.57)	
Highest level of education	No education	284 (7.76)	0.00
	Primary	401 (24.40)	
	Secondary	507 (15.60)	
	Higher	139 (6.66)	
Frequency of reading newspaper	Not at all	797 (9.36)	0.00
	Less than once a week	241 (8.79)	
	At least once a week	197 (6.95)	
	Almost everyday	96 (7.00)	
Frequency of listening to the radio	Not at all	127 (6.75)	0.02
	Less than once a week	132 (9.36)	
	At least once a week	299 (9.11)	
	Almost everyday	773 (8.69)	
Frequency of watching television	Not at all	516 (8.78)	0.02
	Less than once a week	229 (9.74)	
	At least once a week	264 (8.95)	
	Almost everyday	322 (7.57)	
Currently working	No	79 (3.00)	.00
	Yes	1252 (9.71)	
Covered by health insurance	No	1297 (8.50)	0.91
	Yes	34 (8.50)	
Wealth index	Poorest	278 (8.90)	0.02
	Poorer	253 (8.80)	
	Middle	286 (9.30)	
	Richer	296 (8.00)	
	Richest	218 (7.10)	

**Table 5:** Factors influencing tobacco use across different SES groups.

Socio-demographic factors	Q1 (Poorest) N = 278	Q2 (Poorer) N = 253	Q3 (Middle) N = 286	Q4 (Richer) N = 296	Q5 (Richest) N = 218	p-value
**Religion**						<0.001
Christian	135 (17.0)	132 (16.6)	160 (20.2)	202 (25.4)	165 (20.8)	
Islam	120 (25.6)	108 (23.0)	109 (23.2)	82 (17.5)	51 (10.7)	
Traditional	21 (36.8)	12 (21.1)	14 (24.6)	9 (15.8)	1 (1.7)	
Other	1 (12.5)	1 (12.5)	2 (25.0)	3 (37.5)	1 (12.5)	
**Literacy**						<0.001
Cannot read at all, blind, visually impaired	163 (39.18)	115 (27.64)	85 (20.43)	46 (11.06)	7 (1.68)	
Able to read only parts of sentence	33 (21.29)	36 (23.23)	38 (24.52)	36 (23.23)	12 (7.74)	
Able to read whole sentence	78 (10.48)	101 (13.58)	158 (21.24)	212 (28.49)	195 (26.21)	
No card with required language	2 (22.22)	1 (11.11)	2 (22.22)	2 (22.22)	2 (22.22)	
**Level of education**						<0.001
No education	134 (47.18)	79 (27.82)	46 (16.20)	19 (6.69)	6 (2.11)	
Primary	83 (20.72)	102 (25.43)	100 (24.85)	90 (22.40)	26 (6.60)	
Secondary	57 (11.24)	63 (12.40)	122 (24.20)	147 (28.86)	118 (23.30)	
Higher	4 (2.88)	9 (6.47)	18 (12.95)	40 (28.78)	68 (48.92)	
**Read news paper**						<0.001
Not at all	226 (28.57)	195 (24.65)	175 (22.12)	137 (17.32)	58 (7.33)	
Less than once a week	36 (15.06)	38 (15.90)	61 (25.52)	60 (25.10)	44 (18.41)	
At least once a week	14 (7.18)	12 (6.15)	40 (20.51)	73 (37.44)	56 (28.72)	
Almost everyday	1 (1.08)	5 (5.38)	8 (8.60)	22 (23.66)	57 (61.29)	
**Listens to radio**						<0.001
Not at all	60 (47.62)	33 (26.19)	20 (15.87)	9 (7.14)	4 (3.17)	
Less than once a week	40 (30.30)	26 (19.70)	30 (22.73)	24 (18.18)	12 (9.09)	
At least once a week	69 (23.15)	57 (19.13)	66 (22.15)	58 (19.46)	48 (16.11)	
Almost everyday	108 (14.01)	137 (17.77)	169 (21.92)	204 (26.46)	153 (19.84)	
**Watch TV**						<0.001
Not at all	212 (41.25)	150 (29.18)	100 (19.46)	47 (9.14)	5 (0.97)	
Less than once a week	43(18.86)	49 (21.49)	71 (31.14)	44 (19.30)	21 (9.21)	
At least once a week	16 (6.08)	37 (14.07)	75 (28.52)	87 (33.08)	48 (18.25)	
Almost everyday	7 (2.18)	14 (4.36)	40 (12.46)	117 (36.45)	143 (44.55)	

### Proportion of households that use tobacco

A total of 1787 (5.25%) of the households surveyed used tobacco in any form. Out of these households that used tobacco, 186 (10.40%) were in the urban areas while 1601 (89.60%) were in the rural areas. The proportion of households that use different forms of tobacco was disaggregated by type of place of residence (urban and rural) Cigarette smoking and using of snuff are the predominant forms of tobacco use in both rural and urban households. (Table 2).

### Individual tobacco use among male household members

Tobacco use among male household members was disaggregated by the different forms in which tobacco is consumed in Nigeria. Out of the 15,486 individual male household members studied, 1847 (11.90%) used tobacco. When disaggregated by the different forms of tobacco use, the prevalence of cigarette smoking was the highest, 1331 (8.59%), while chewing of tobacco was the least, 100 (0.65%). (Table 2)

### SES differences among individual males that use tobacco

The richest socio-economic group use tobacco less (8.74%) than the poorest socio-economic group (13.40%) at a statistically significant level of p<0.05 (Table 2)

### SES differences in cigarette use and expenditure among male household members

The highest proportions of the respondents that smoke cigarettes are in the middle and richer socio-economic groups, followed by the poorest and poorer groups. Those in the richest SES group smoked cigarettes the least.

Across SES, there were significant differences in the number of sticks of cigarettes smoked per day (p<0.05). The proportion of respondents across all the SES groups that smoke less than 9 sticks of cigarette per day is greater in the rich SES group in comparison to the poor SES group.

Over 75% of respondents in all SES groups appear to spend less than 0.60USD per day on cigarettes, there is a statistically significant difference in spending across the SES quintiles. A greater proportion of male household members in the richer SES groups spend <0.60USD/day on cigarettes compared to those in the poorer groups who spend between 0.60–1.19USD/day. (p < 0.05) (Table 3)

The mean and median daily consumption of cigarette is 5.80 (± 6.47) and 4.00 USD respectively

The mean and median daily cost of cigarette is 2.96 (± 15.79) and 0.25USD respectively

### Socio-demographic factors associated with cigarette smoking among individual males

The socio-demographic and SES factors associated with cigarette smoking among male household members are presented in Table 4 above. Significant association is found between cigarette smoking and religion, literacy, level of education, employment, wealth index and frequency of reading newspapers, listening to radio and television (p < 0.05). The association between cigarette smoking and type of place of residence or being covered by health insurance were not statistically significant.

### Factors influencing tobacco use across different SES groups

The influence of religion on tobacco use varies across the SES groups. Islam influences tobacco use more in the poor SES groups, while Christianity influences it more in the rich SES groups.

Respondents in the poor SES groups who cannot read at all and have no education use more tobacco than their counterparts in the rich SES groups. However those with secondary school education in the richer SES seem to use more tobacco than the others. Among those exposed to both print and electronic media, respondents in the rich SES groups read newspapers more often than the poor groups and the rich SES groups watch TV more than the poor. (All results are < 0.05)

### Binary logistic regression of factors associated with cigarette smoking among male household members

A logistic regression analysis of statistically significant factors associated with cigarette smoking among male household members is presented in Table 6 above. The effect of each predictor variable on cigarette smoking was analyzed independently. There is an inverse relationship between cigarette smoking and being literate, being either a Christian or Muslim, having higher education, reading newspapers, watching television, and being in the richer or richest SES groups. Statistically significant inverse relationships are seen only with literacy, reading newspapers and belonging to the richest SES group (p < 0.05). Male household members who are literate are 0.8 times as likely (or 1.25 times less likely) to smoke as those who are not literate; those who read newspapers are 0.79 times as likely (or 1.27 times less likely) to smoke as those who do not; while those in the richest SES group are 0.78 times as likely (or 1.28 times less likely) to smoke as those in the poorest quintile.

**Table 6:** Binary logistic regression of factors associated with cigarette smoking in individual males.

Variables	Standardized coefficient	p-value	Unadjusted OR	Confidence interval		**Adjusted OR
				Lower limit	Upper limit	
**Religion**						
Other	-	<0.01	-	-	-	-
Christian	-0.25	0.52	0.78	0.37	1.65	0.75
Islam	-0.73	0.06	0.48	0.23	1.02	0.41
Traditional	0.93	0.02	2.54	1.41	5.66	2.08
*Literacy	-0.22	<0.01	0.80	0.71	0.91	0.73
**Level of education**						
No education	-	<0.01	-	-	-	-
Primary education	0.51	<0.01	1.67	1.42	1.96	1.44
Secondary education	0.01	0.01	1.01	0.87	1.17	1.00
Higher education	-0.16	0.13	0.85	0.69	1.05	0.92
*Reads newspaper	-0.23	<0.01	0.79	0.71	0.89	0.89
*Listens to radio	0.28	0.03	1.33	1.01	1.61	1.59
*Watches television	-0.03	0.59	0.99	0.86	1.01	0.98
**Wealth index (SES group)**						
Poorest	-	0.02	-	-	-	-
Poorer	-0.02	0.06	0.98	0.82	1.17	0.92
Middle	0.05	0.31	1.05	0.88	1.25	0.99
Richer	-0.01	0.01	0.99	0.84	1.18	0.97
Richest	-0.25	0.01	0.78	0.65	0.94	0.81
**Constant**	-2.01	0.00				0.13

*Predictors coded as ***yes = 1*** and ***no = 0*.** Reference categories are ‘***not literate’; ‘does not read newspaper’; ‘does not listen to radio’;’ does not watch television’***. Reference categories—for Religion is ‘other’; for wealth index ‘poorest’; and for level of education, ‘no education’

**All other variables were held constant

There is a direct relationship between cigarette smoking and being a traditionalist, having only primary or secondary education, listening to radio, being currently employed, and belonging to the middle SES quintile. Statistically significant relationships are found with being currently employed, having only primary education, listening to radio and being a traditionalist. Male household members who smoke cigarette are 3.47 times more likely to be currently working, 2.54 times more likely to be traditionalist, 1.67 times more likely to have only primary education and 1.33 times more likely to listen to radio than those who do not smoke cigarette.

## Discussion

The proportion of individual male household members who use tobacco appear to be more than the proportion of households that consume tobacco. A possible explanation for this is the fact that there could be more than one male household member using tobacco in each household. The prevalence of tobacco use among male household members obtained from this study is similar to the WHO reported prevalence of 13% for males in Nigeria [8]. Though this figure is less than what was observed in other developing countries like Malawi (23.7%) and Tanzania (24.8%) [8], it translates to more number of people in real terms as the population of Nigeria is much larger than the population of these other countries.

Tobacco consumption in high-income countries appear to have been decreasing over the past two decades while consumption in developing countries has been found to be increasing by 3.4% annually [9]. The pressures of large multinational tobacco companies who try to, and often succeed in entering developing countries have led to a corresponding rise in tobacco consumption. These tobacco companies have found in some developing countries, an emerging under-regulated market for exploitation [9]. In Nigeria for instance, the continued active presence of major tobacco companies in the country and non-implementation of the tobacco control policy means that the reported rate could rise to epidemic proportions if deliberate steps are not taken towards its control.

The urban-rural disparity in tobacco consumption observed in this study corroborates findings from Rani et al’s study in India, which reported higher levels of tobacco use among households in the rural areas [10]. It is also worthy to note that apart from cigarette smoke, smokeless tobacco in the form of snuff (which is usually inserted in the nostrils and inhaled) is used by a lot of the respondents. This could be as a result of the fact that in most cultures in rural Nigeria, smokeless tobacco is not perceived to be harmful and is used for certain traditional functions and cultural rites such as traditional wedding ceremonies, where it is an honorable gift to the in-laws. Cultural myths around smokeless tobacco may also explain the higher prevalence rate in rural areas. Such myths include: for example ‘snuffing tobacco clears upper respiratory tract infections; chewing tobacco aids digestion of food’. Such cultural practices could further increase the ill effects of tobacco use, for example nasopharyngeal carcinomas. The awareness of the ill effects of smoking or tobacco use is concentrated in the urban areas, leaving people in the rural areas with inadequate knowledge of the dangers of tobacco use to health. The wide disparity in tobacco use between the urban and rural dwellers could also be as a result of delayed access to medical and media resources that highlight the ill effects of tobacco use. It could also be attributed to low educational attainment by the rural residents which limit their understanding of written anti-tobacco messages [11].

The prevalence of cigarette smoking was found to be higher than all other forms of tobacco use. Recent surveys in Nigeria have reported similar cigarette smoking prevalence rates, with values that have ranged between 7.2% and 10.5% [4, 12]. Low and middle-income countries (LMIC) account for 82% of cigarette smokers worldwide and this could be explained by the aggressive marketing of cigarettes relative to other forms of tobacco by multinational tobacco companies [13]. Cigarette smoking has been identified as a leading cause of preventable disease and premature death in industrialized countries and it is projected that by 2030, 70% of deaths due to tobacco will occur in developing countries [14]. This notwithstanding, the tobacco industry continues to successfully influence governments of LMICs to delay the approval and/or implementation of regulations to control smoking, and loosen any restrictions on advertising cigarettes [9].

Several determinants of smoking which have been promulgated in theory include: demographics such as age, gender, marital status, SES; situational factors such as job strain; and intrapersonal and smoking outcomes in others [15]. This study however was only able to explore some of these factors having used secondary data. High level of literacy was found to be significantly associated with lower rates of cigarette consumption. A strong negative relationship was also seen between the highest level of education and cigarette smoking, implying that education reduces the likelihood that a person would smoke cigarettes. These associations could be explained by the fact that most tobacco control adverts in Nigeria are written and only those who are able to read are being reached. Again, it is known that education exposes people to information, including anti-tobacco campaign messages, and those who are exposed are most likely to be aware of the negative health effects of tobacco products which will possibly induce a change in behavior [13, 16, 17]. This association between smoking and lack of education has been reported in other studies conducted in LMICs [13, 16].

Income was seen to have a positive association with cigarette smoking, as those who are currently employed, irrespective of their place of residence, consumed more cigarettes than their unemployed counterparts. However, the proportion of those who smoked was quite high among the poorest SES group. This association with poverty has also been seen in other studies [13, 16] but it was interesting to find in our study that the larger proportion of respondents who smoked cigarettes were in the middle and richer SES groups when compared with other SES groups. This association could be explained by the ability to pay for tobacco products among these two SES groups or it could be a reflection of the fact that there are factors besides socioeconomic status which account for the use of cigarettes. The richest SES quintile on the other hand appeared to smoke fewer cigarettes and consequently spent less on tobacco than the other quintiles. A combination of socio-demographic factors that expose these respondents to various anti-smoking campaigns, policies and laws could explain this.

As earlier stated, tobacco use in general which encompasses both smoke and smokeless tobacco was found to be higher in the poorest than the richest group. The poorer SES groups smoke more sticks of cigarette in a day, and also spend a greater fraction of their income on cigarettes. This excludes the huge cost that will be incurred from the impact of tobacco on their health. The choice to smoke cigarettes by poor people has been attributed to lack of awareness about adverse effects of smoking and the tendency to take up smoking as a coping mechanism for poverty-related stress. While this may temporarily distract from the stress of poverty, it worsens the situation, since spending significant amounts of income on tobacco depletes available resources to meet the basic human needs of food, shelter, healthcare and education.

According to Mushtaq et al., a 10% increase in cigarette prices could decrease consumption by 11.7% [18]. This decrease might be greater among the poor who are considered to be more responsive to increase in price of commodities [19]. However increasing tobacco taxes alone may not be sufficient to achieve a reduction in tobacco consumption. A consolidated approach such as the World Health Organization (WHO) MPOWER package needs to be implemented to achieve this reduction. This package incorporates six policies that countries and organizations need to enforce in combination with their established anti-tobacco mechanisms, to combat tobacco use: monitor tobacco use and prevention policies; protect people from tobacco smoke; offer help to people who want to quit tobacco use; warn about dangers of tobacco; enforce bans on tobacco advertising, promotion and sponsorship; and raising tobacco taxes. It is stated that targeting these policies at everyone, with special efforts made to reach the poor and uneducated, could potentially lead to reduction in the prevalence of smoking and tobacco consumption on the whole.

## Conclusion

The proportion of households that use tobacco in Nigeria is relatively low when compared to other countries, however the translation of this percentage of households to numbers in the Nigerian population yields significantly higher numbers than other LMICs. Various factors influence cigarette smoking in Nigeria, namely: SES, educational level, literacy and traditional or cultural practices. The poorer SES groups smoke more and spend more purchasing cigarettes than the richest SES group, but poverty alone does not influence smoking of cigarette or using tobacco products. Thus legislation or the implementation of the tobacco control bill alone may not effectively reduce the use of tobacco and its products in Nigeria. The WHO MPOWER package and other strategies that incorporate behavioral change, management procedures targeted at all groups of people—poor and rich, educated and uneducated, urban and rural residents—may produce better results.
